# Effects of 10 Weeks of Walking With Mobile Step-Tracking Apps on Body Composition, Fitness, and Psychological State in Adolescents Who Are Overweight and Obese: Randomized Controlled Trial

**DOI:** 10.2196/55243

**Published:** 2024-12-10

**Authors:** Adrián Mateo-Orcajada, Cristina M Ponce-Ramírez, Lucía Abenza-Cano, Raquel Vaquero-Cristóbal

**Affiliations:** 1 Facultad de Deporte Universidad Católica de Murcia (UCAM) Murcia Spain; 2 Research Group Movement Sciences and Sport (MS&SPORT) Department of Physical Activity and Sport, Faculty of Sport Sciences University of Murcia Murcia Spain

**Keywords:** adolescents, obesity, physical activity, overweight, mobile app, physical education

## Abstract

**Background:**

In recent decades, physical activity intervention programs have been developed to reduce overweight and obesity in adolescents. However, this population is considered hard to reach in physical activity programs due to lack of adherence and poor results. Interventions with mobile phones in the adolescent population with normal weight have shown benefits, so this line of research may provide benefits in adolescents with overweight or obesity, although it has not yet been explored in the scientific literature.

**Objective:**

This study aims to determine the changes produced by a 10-week intervention promoted during school lessons on physical education using step tracker mobile apps in out-of-school hours on physical activity, adherence to the Mediterranean diet, body composition, and the physical condition of adolescents who are overweight and obese, and to analyze the changes achieved by the 10-week intervention on the psychological state of adolescents who are overweight and obese.

**Methods:**

The study was based on a randomized controlled trial with an initial sample of 50 adolescents aged between 12 and 16 years (from the first to the fourth years of compulsory secondary education), whose body composition, physical activity level, physical condition, and psychological state were measured. Participants were divided into an experimental group (EG) and a control group (CG), where the EG performed a series of walking steps with a mobile app in their free time outside physical education classes. Adolescents in the CG continued to perform their physical activities as normal but did not use any mobile apps. Inclusion in the EG and CG was randomized, and the researchers were blinded.

**Results:**

An increase was found in the EG in corrected arm girth (mean difference –0.46; *P*=.05), curl-up repetitions (mean difference –6.35; *P*=.02) and push-up repetitions (mean difference –2.27; *P*=.04) after the intervention. In the CG, there was a significant increase in hip girth (mean difference –1.37; *P*=.05), corrected thigh girth (mean difference –1.28; *P*=.04), and muscle mass (mean difference –0.87; *P*=.04), as well as a significant decrease in competence (mean difference 3.08; *P*=.03). The covariates gender and age showed an effect on corrected arm girth (gender: *P*=.04), curl-up repetitions (gender: *P*=.04) and push-up repetitions (gender: *P*=.04) in the EG; while in the CG it affected corrected thigh girth (gender: *P*=.04), adherence to the Mediterranean diet (gender: *P*=.04 and age: *P*=.047) competence (gender: *P*=.04 and age: *P*=.04) and relatedness (gender: *P*=.05 and age: *P*=.04). No significant differences were found when comparing changes in the CG and EG.

**Conclusions:**

A 10-week program of mobile app use by adolescents who are overweight and obese for physical activity outside of school hours does not appear effective in producing improvements in body composition, physical fitness, or adequate psychological state as it does not appear to significantly increase physical activity.

**Trial Registration:**

ClinicalTrials.gov NCT06089876; http://clinicaltrials.gov/ct2/show/NCT06089876

## Introduction

### Development and Prevalence of Overweight and Obesity in Childhood and Adolescents

Overweight and obesity, understood as an accumulation of excessive fat, are the second leading cause of preventable and avoidable mortality in countries without limited resources [1], with >340 million children and adolescents affected by this disease worldwide [1]. This pathology increases the risk of experiencing other chronic diseases at an early age and during adulthood, such as arterial hypertension [2], diabetes [3], or various types of cancer [4]. This pathology increases the probability of experiencing psychological disorders, such as stress, depression, and anxiety [5], and it decreases life expectancy [6]. Because of the reasons mentioned earlier, overweightness and obesity at an early age are already considered by the World Health Organization (WHO) as a pandemic [7]. This is because it has a high incidence in countries without limited resources and especially in Spain, a country that has been at the top of the rankings of childhood obesity in the last decade, leading them on some occasions [8-10].

### Factors Influencing the Development of Overweight and Obesity in Childhood and Adolescents

This worrying situation was compounded by the COVID-19 pandemic. The period of confinement that took place in most countries brought with it a series of changes in the lifestyle habits of adolescents, such as the impossibility of engaging in physical-sports activities [11] and the increased use of technological devices [12,13]. Although the restrictions caused by the COVID-19 pandemic have been lifted, the lifestyle habits generated during the pandemic have become established in the postpandemic adolescent population. More specifically, the level of physical activity of prepandemic adolescents has not recovered [14]. More than 80% of adolescents aged between 12 and 16 years old do not meet daily physical activity recommendations [14], performing <60 minutes per day of aerobic physical activity at moderate or vigorous intensities [15]. This becomes even more relevant when considering that adolescence is a critical stage with respect to the practice of physical activity, with a high rate of sports abandonment during this period [16]. This results in an increase in the percentage of adolescents whose weekly physical activity is limited to that performed in physical education sessions within the school context [17].

In addition, the abuse of new technologies during the COVID-19 pandemic has remained at worrying levels in the post–COVID-19 period [18], favoring an increase in the adolescent population that is considered sedentary [19]. Sedentary behavior is defined as a waking behavior characterized by energy expenditure of <1.5 metabolic task equivalents while maintaining a sitting or reclining posture [20]. The main problem with sedentary behavior lies in the negative health consequences for adolescents [21]. This becomes even more relevant when considering that even if adolescents comply with the physical activity recommendations of 60 minutes per day, they can still experience the negative effects of sedentary behavior if they accumulate many hours per day in sedentary activities [22]. In this sense, male participants spend 507 minutes a day on average in sedentary activities, while female participants spend 523 minutes a day on average, and this is even more pronounced on weekend days [23]. Furthermore, it has also been found that up to 93.8% of adolescent boys and 87.2% of adolescent girls spend >2 hours a day in front of a screen [13]. Mobile phone use has been shown to be a determining factor, with 10% to 16% of adolescents showing problematic use of this device. This is understood as a dependence or need to be connected to the device, as well as difficulties in disconnecting or setting limits on the time dedicated to its use, which interferes with health, well-being, and leisure time behaviors [24,25].

### Impact of Unhealthy Habits on Adolescent Health

The decline in physical activity is all the more significant because it is related to the worsening of other healthy lifestyle habits, such as diet, with adolescents who were less physically active having poorer adherence to patterns, such as the adherence to the Mediterranean diet (AMD) [26,27]. This becomes relevant when it is known that AMD is associated with a reduction in cardiovascular risk of up to 38% [28] and a reduction in the risk of type 2 diabetes mellitus of 19% [29]. Moreover, increased adherence significantly reduces all-cause mortality, as well as the incidence of cancer, Parkinson disease, and Alzheimer disease [30]. Therefore, AMD, as well as the practice of physical activity, plays a decisive role in reducing the prevalence of noncommunicable diseases [31].

However, in recent years, there has been an increase in sedentary time among adolescents and a decrease in physical activity, which, in combination with other unhealthy habits, has led to a greater accumulation of body fat [32], increasing the likelihood of being overweight or obese [33]. Thus, following COVID-19, an increase in adolescents who are overweight and obese has been observed, with increases in some countries of up to 30% and 40%, respectively, making obesity 15% more prevalent in this population than before the COVID-19 pandemic [34]. During the pandemic, there was also a notable increase in psychological disorders affecting the adolescent population [35] mainly due to the restriction of sports practice, which is very beneficial for maintaining an adequate state of mental health [36]. In addition, the absence of social relationships with peers [37], the uncertainty generated regarding the near future, and the abusive use of new technologies [35], also contributed to this. Although the adolescent population in general was affected, adolescents who are overweight and obese were particularly affected, increasing the prevalence of disorders such as depression and anxiety [5].

### Physical Activity Practice and Interventions in Overweight and Obese Children and Adolescents

It should be added that in Spain, only two 1-hour sessions of curricular physical education classes per week are provided [38]. This means that on many occasions the real practice time during the sessions is 45 minutes, in which barely 13% of students who are overweight and obese reach a moderate or vigorous intensity of practice during the sessions [39], making physical and psychological benefits more difficult to achieve. This is true even though schools are a place where adolescents spend a large part of their day [40]. In this context, some studies have sought to encourage the practice of physical activity within the school context in the population that is overweight and obese. More specifically, interventions have been carried out during school hours using gymnasium exercises, and improvements have been observed in cardiovascular fitness [41], as well as in the reduction of body fat and insulin levels in adolescents who are obese [41].

In addition, more recent studies have used high-intensity interval training (HIIT) 3 times a week within the school setting, achieving beneficial effects on adolescent girls who are overweight [40]. In this regard, these school-based interventions have shown benefits in body weight, waist-hip ratio, fat percentage, and aerobic capacity in adolescents who are overweight [42,43]. Therefore, research supports that school-based interventions are highly effective in improving body composition in the populations that are overweight and obese [40,41].

However, these interventions also have their detractors, who argue that physical education hours are too limited to devote so much time to a single content and that the pedagogical component is also overlooked during implementation [44,45]. As an alternative to this, it has been suggested that physical education classes could promote interventions that encourage adolescents to practice physical activity in their free time [19]. This way, they would be encouraged to walk a minimum weekly distance by means of mobile apps that allow them to monitor the activity carried out, which is also an element that generates adherence and enjoyment in this population [46]. In this regard, previous studies have shown that the use of a physical activity program controlled by mobile step tracker apps in out-of-school hours has provided benefits in increasing the level of physical activity and improving body composition in adolescents [19,47], as well as in the psychological state of this population [35].

### Mobile Apps as Promoters of Physical Activity in Overweight and Obese Children and Adolescents

Despite the benefits obtained with the use of mobile apps in the general adolescent population [19], the use of mobile apps by adolescents who are overweight and obese shows contradictory results, and no accurate conclusions can be firmly drawn [48]. This is because the scientific literature on the use of mobile devices to increase physical activity is scarce in this population, as they have been historically considered a “hard-to-reach population” [49]. Perhaps this is the reason why no previous research has been found on adolescents who are overweight and obese using mobile apps to promote compulsory leisure-time physical activity in physical education classes [19]. However, recent research has shown that there are no differences in how adolescents rate these physical activity apps based on their weight status, nor are there differences in their problematic use of these apps [50], so they could be an effective resource for adolescents who are overweight and obese as well.

Therefore, the objectives of the present research were (1) to determine the changes produced by a 10-week intervention promoted by the physical education school subject using step tracker mobile apps in out-of-school hours on physical activity, AMD, body composition, and physical condition of adolescents who are overweight and obese; and (2) to analyze the changes achieved by the 10-week intervention on the psychological state of adolescents who are overweight and obese.

Given the objectives of this research and the scarce previous research conducted with mobile apps for physical activity in adolescents who are overweight or obese, it is not possible to posit consistent research hypotheses, but it is expected that the promotion of the use of mobile apps from the subject of physical education will lead to significant benefits in the level of physical activity, favoring changes in body composition, greater AMD, and improved physical condition in this population (hypothesis 1); and that increased physical activity has a positive effect on the psychological state of adolescents who are overweight and obese (hypothesis 2).

## Methods

### Design

A randomized controlled trial was conducted in which adolescents were divided into a control group (CG) and an experimental group (EG). Adolescents in the EG used the STRAVA (Strava, Inc) mobile app for physical activity for 10 weeks, a minimum of 3 times per week, to walk the indicated distance, which was incrementally increased as the weeks passed.

The CONSORT (Consolidated Standards of Reporting Trials) guidelines were followed for the research design, and the study was registered before commencement on ClinicalTrials.gov (NCT06089876). The institutional ethics committee of the Catholic University of Murcia approved the study design in accordance with the World Medical Association and following the Declaration of Helsinki (CE022102).

A school in the region of Murcia with the highest number of adolescents in compulsory secondary education of an urban locality was selected. The school with the largest number of adolescents was contacted, and if the management was not interested in participating, the next school with the largest sample was contacted. Once the school was chosen, contact was made with the school’s management team. Once approval was obtained, data collection was coordinated with those in charge of the physical education department. Subsequently, a meeting was held with the adolescents and their parents to explain the purpose and procedure of the study, emphasizing confidentiality in the treatment of the data obtained. Those who agreed to participate provided an informed consent signed by them and their parents before the start of the research.

### Participants

The sample size calculation was performed using the statistical software Rstudio (version 3.15.0; Rstudio Inc). For this purpose, the SDs of previous research on the use of mobile apps by adolescents to increase physical activity were used (SD 0.68) [19]. Thus, for an error (d) of 0.27 and a 95% CI, the minimum sample needed for each group was 25 adolescents [51].

**Figure 1:**
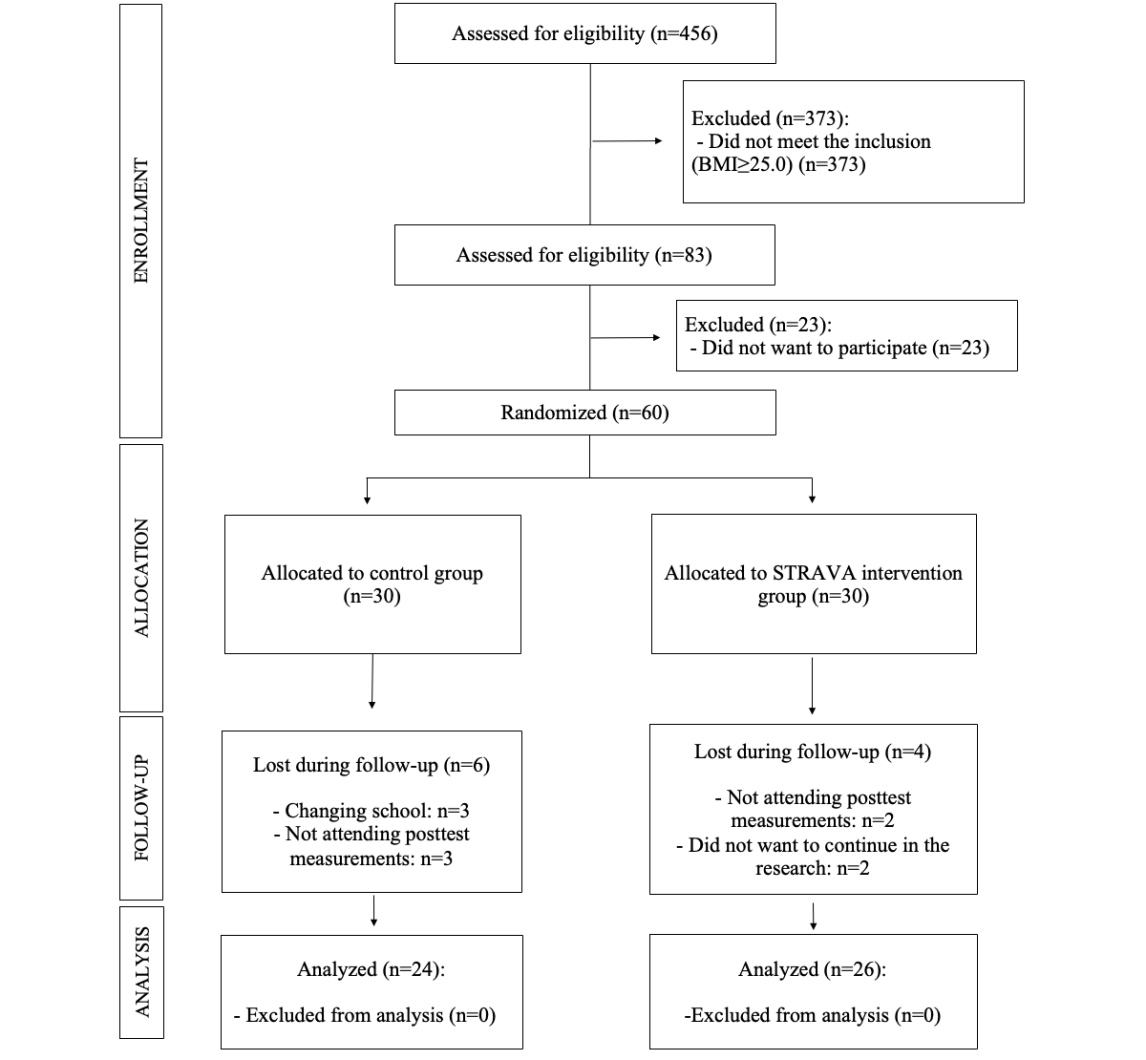
Flow diagram of the sample selection.

A total of 456 adolescents were enrolled in the school, of whom 83 (18.2%) were overweight or obese. The initial participation in the research was 60 adolescents, of whom 50 (83%) completed the research (n=26, 52% EG and n=24, 48% CG; Figure 1). A total of 24 male participants (n=11, 46% in the EG and n=13, 54% in the CG; mean age at peak height velocity [APHV] 13.09, SD 0.79 years) and a total of 26 female participants (n=15, 58% in the EG and n=11, 42% in the CG; mean APHV 12.45, SD 0.69 years) participated in the research. The age range was between 12 and 16 years (mean 14.11, SD 1.24 years), and all had a BMI ≥25 (mean BMI 27.66, SD 2.30 kg/m^2^; total overweight: n=40; total overweight in the EG: n=21; total overweight in the CG: n=19; total obese: n=10; total obese in the EG: n=5; and total obese in the CG: n=5).

Although BMI has limitations in its use, such as lack of sensitivity in the analysis of fat and fat-free mass, as well as in distinguishing between overweight and obesity in children [52-54], it also has positive factors that make it used ahead of other methods. Simplicity of use is one of its main characteristics [55,56]; when measured accurately and in accordance with growth charts, it is a reliable indicator of overweight and obesity [52,55]; it is a predictive factor that is strongly associated with adult obesity and overweight [53,57]; and the other alternatives have also shown no greater sensitivity than BMI for detecting overweight and obesity in child and adolescent populations [55,57,58].

The inclusion criteria for individuals were as follows: (1) age between 12 and 16 years, (2) compulsory secondary education, and (3) a BMI ≥25 kg/m^2^. The exclusion criteria were (1) adolescents in the intervention group not having their own mobile phone; and (2) starting any regular physical activity that was not practiced before the start of the research, understood as going to the gymnasium or starting a specific sport, in both CG and EG.

### Randomization and Blinding

The randomization was performed by the principal investigator using a computer-generated random number table in the presence of other investigators not participating in the research. The randomization was carried out considering the BMI of the adolescents. Students were randomly assigned to the CG and EG.

Preintervention measurements were performed after the randomization process. The researchers in charge of the measurements were blinded to the group to which each adolescent belonged. In the postintervention measurements, the researchers were not aware of the scores obtained by each adolescent in the preintervention measurement. In addition, the researchers who oversaw the monitoring of the distance traveled during the intervention by each adolescent were not aware of the scores obtained by each adolescent during the preintervention measurement.

### Instruments

#### Physical Activity Level, AMD, and Psychological State

To assess the level of physical activity of adolescents who are overweight and obese, the Physical Activity Questionnaire for Adolescents (PAQ-A) [59] was used. This is a validated questionnaire in Spanish with moderate validity and reliability (intraclass correlation coefficient of 0.71 for the overall score of the same questionnaire) [60]. The questionnaire consists of 9 items, of which the first 8 are completed using a Likert scale of 1 to 5 points (1: no physical activity and 5: high physical activity), and the last item is answered dichotomously (yes or no). The final score is calculated by the arithmetic mean of the first 8 items (with a minimum score of 1 and a maximum of 5). A higher score reflects a greater engagement in physical activity [59].

AMD was assessed using the Mediterranean Diet Quality Index (KIDMED) [61]. This questionnaire consists of 16 items that are scored as 1 (meets the criteria) or 0 (does not meet the criteria). Out of the 16 items, 12 have a positive connotation (meeting the criterion contributes +1), while the other 4 have a negative connotation (meeting the criterion contributes –1). The final score ranges from 0 to 12 points, with a higher score indicating a greater AMD. The KIDMED has moderate validity and reproducibility for use in adolescents (α=0.79; κ=0.66) [61].

The Basic Psychological Needs Scale (BPNS) [62] and the Satisfaction With Life Scale (SWLS) [63] were used to assess the psychological state of adolescents. The BPNS consists of 18 items distributed in 3 dimensions (competence, autonomy, and social relatedness). Each dimension is composed by 6 items rated on a Likert scale from 1 to 6 points, so the final score for each dimension ranges from 6 to 36 points, with a higher score indicating higher satisfaction; the SWLS has 5 items rated on a Likert scale from 1 to 5 points, with the final score ranging from 5 to 25 points [64]. A higher score on the SWLS shows a higher life satisfaction. Both scales have been previously validated for use in adolescents [65], showing adequate validity and internal consistency (α=0.80 for competence, α=0.69 for autonomy, and α=0.73 for social relatedness) [64,65].

#### Kinanthropometric and Body Composition Measurements

The body composition analysis was performed following the protocol established by the International Society for the Advancement of Kinanthropometry (ISAK) [66]. Two accredited anthropometrists (levels 3 to 4) measured adolescents who are overweight and obese. The measurement consisted of 2 basic measurements (body mass and height), skinfold in 3 areas (triceps, thigh, and calf) and girth in 5 areas (arm relaxed, waist, hips, thigh, and calf).

Body mass was measured using a TANITA BC 418-MA Segmental scale (TANITA) with an accuracy of 100 g; height was measured with a SECA 213 stadiometer (SECA); girth was measured using Lufkin W606PM inextensible tape (Lufkin) with an accuracy of 0.1 cm; and skinfold was measured using a caliper with an accuracy of 0.2 mm (Harpenden). Before the pre- and postintervention measurements, all instruments were calibrated.

In total, 2 measurements were made for each variable, and if the difference between the 2 measurements was >5% in skinfold or 1% in all other measurements, a third measurement was made. When 2 measurements were taken, the final value corresponded to the average of the 2 measurements. However, when a third measurement was included, the final value was the median of the 3 measurements [66].

The intra- and interrater technical errors of measurement (TEMs) were calculated on a subsample. The intrarater TEM was 0.04% for basic measurements, 1.58% for skinfold, and 0.06% for girth. The interrater TEM was 0.06% for basic measurements, 2.01% for skinfold, and 0.08% for girth.

With the final values of each of the anthropometric measurements, the following variables were calculated: BMI [67], fat mass (%) [67], muscle mass (%) [68], sum of skinfold in 3 areas (∑3; triceps, thigh, and calf) [63], waist-height ratio (waist girth/height), and corrected girth of the arm (arm relaxed girth–[π×triceps skinfold]), thigh (middle thigh girth–[π×thigh skinfold]), and calf (calf girth–[π×calf skinfold]).

The maturity offset was estimated using the sex-specific formula by Mirwald et al [69]. This method has shown validity for estimating maturity offset against the gold standard (radiograph of the left wrist), with *R*^2^ values of 0.92 to 0.89 for male participants and 0.91 to 0.88 for female participants. The result was used to calculate the APHV by means of the following formula: APHV = chronological age – maturity offset. APHV is defined as the age at which there is a dramatic increase in the rate of growth in height and body mass in adolescents [70]. Therefore, a positive value in the maturity offset indicated how many years ago that participant had passed his or her APHV, while a negative value indicated the years remaining before that adolescent reached APHV [69].

#### Physical Fitness Test

The physical condition of the adolescents was assessed according to previous research carried out in this population [19,71]. The physical capacities assessed were cardiorespiratory capacity, hamstring flexibility, upper limb strength, lower body explosive power, speed, and abdominal strength endurance.

Cardiorespiratory capacity was measured using the 20-m shuttle run test [72]. This is a maximal incremental test where 20 m must be run as many times as possible before the beep sounds. The test is terminated when the participant is exhausted or is unable to complete the distance before the beep sounds on 2 consecutive occasions [19]. At the end of the test, the maximal oxygen uptake can be calculated using the formula by Léger et al [72].

For hamstring flexibility, the sit-and-reach test was used [73]. For its correct execution, the adolescents start seated with their legs extended and ankles flexed at 90°, allowing their feet to rest against an Acuflex Tester III box (Novel Products). From this position, the participant must reach the maximum possible distance by moving the palms of the hands, one on top of the other, along the box by pushing a bar, keeping the legs straight and the feet fully supported against the box at all times [74].

For upper limb strength, the handgrip strength test and the push-ups test were used. Handgrip strength consisted of applying the maximum possible force on a Takei TKK5401 portable digital dynamometer (Take Scientific Instruments) with the elbow fully extended [75]. This is because this position has been shown to be the most valid for applying maximal force. This test has been previously validated for use in this population [76] and was performed on both the right and left hand [77]. For the push-up test, the participants had to start in the prone position with only their feet and hands in contact with the ground. The hands were placed at the sides of the body with the elbows bent at 90°, while the tip of the feet was the area in contact with the ground. Once in the starting position, the adolescents had to push up from the floor with their backs and legs fully straight, achieving a full extension of the arms. The test ended when the adolescents could not fully extend their arms or when 1 minute was exceeded. The final value corresponded to the maximum number of correctly executed repetitions [78].

Lower limb explosive power was assessed using the countermovement jump (CMJ). The adolescents started standing on a force platform (MuscleLab), with hands on hips and feet hip-width apart. From this position, the maximum possible height was to be reached by performing a vertical jump. To do this, the adolescents performed a 90° knee flexion followed by a maximum knee extension without stopping between the 2 phases, keeping their hands on their hips and as vertical as possible during the flight phase. The knees and ankles had to be fully extended during the flight phase [79].

The 20-m sprint test was used to measure the speed of the adolescents. During the test, the adolescents had to cover 20 m in the shortest possible time [80]. Single-beam photocells (Polifemo Light Microgate), located at hip height, were used to measure the time it took the adolescents to cover the distance [81], as this arrangement has been shown to give the greatest validity and reliability to the test. In this position, there is only a 4% chance that the arms will cut the photocell before the rest of the body, whereas at chest height the probability of this happening increases to 60% [82].

Abdominal strength endurance was measured using the curl-up test. The adolescents were placed in supine position on a mat with their arms supported and crossed over their chest, their knees bent at 90°, and their feet fully supported on the floor. From this position, the participant had to perform the maximum number of trunk flexions, lifting the upper back off the ground, until exhaustion was reached or 1 minute of time had elapsed [83].

### Procedure

First, the PAQ-A, KIDMED, BPNS, and SWLS questionnaires were completed. Subsequently, the anthropometric assessment was carried out by the ISAK-accredited anthropometrists. Once this was completed, the physical fitness assessment tests were carried out. Before warming up, all the adolescents performed the sit-and-reach test, as previous research has shown that the warm-up influences the performance obtained in this test [84]. Once this test was completed, the adolescents were given an explanation about the handgrip strength, push-ups, CMJ, 20-m sprint, and curl-up tests to become familiarized with them. The adolescents underwent a 5-minute warm-up consisting of progressive running and mobility of the joints involved in the fitness tests (ankles, knees, hips, wrists, and shoulders). The participants randomly performed 2 repetitions of each of the fitness tests, allowing 2 minutes of rest between repetitions of each test and 5 minutes between the different tests. The best repetition was selected as the final value of the test. Once these physical fitness tests were completed, the adolescents performed a single repetition of the 20-m shuttle run test, preventing the fatigue of this maximum test from influencing the execution of the rest of the tests.

It should be noted that all the measurements were carried out during school hours corresponding to physical education classes. The questionnaires were completed in a classroom at the school, avoiding any distractions and maintaining a calm atmosphere for their correct completion. The anthropometric assessment was carried out in the changing rooms of the sports hall, maintaining a stable temperature and as much privacy as possible during the measurements. The pre- and postintervention measurements were carried out at the same time in all groups, as it was the school time provided for the school physical-education classes, thus avoiding that the changes that occur in the body composition variables during the day could affect the results [85]. The physical fitness tests were carried out in the sports hall, maintaining a stable temperature and preventing polluting atmospheric variables from influencing the results.

The warm-up and execution of the physical condition tests were supervised by researchers with previous experience in the execution of these tests, avoiding possible errors in the execution and collection of data. The order of the tests was determined according to the National Strength and Conditioning Association, which bases its suggestions on the fatigue generated during the tests as well as on the metabolic pathways required for each of them [86].

### Mobile App Intervention

The initial sample of participants (n=60) was divided into the EG (n=30) and CG (n=30), with the EG adolescents using the STRAVA mobile app for 10 weeks after school. The CG did not use any mobile app in out-of-school hours. Both EG and CG continued to attend physical education classes as normal. Pre- and postintervention physical activity level, body composition, psychological state, and physical condition of all participants were measured.

The assignment given to each group was randomized. Before starting the intervention, the physical activity level, body composition, psychological state, and physical fitness of all adolescents were assessed. This was followed by the 10-week intervention. The EG adolescents were required to use STRAVA after school at least 3 times per week, completing a minimum of 4600 steps per day the first week, ending with 10,000 steps per day, as this is the minimum value for the physical activity performed to be considered moderate-vigorous [55,87]. So, this distance was defined as the final target (week 10: 10,000 steps per day), which was reached by increasing the distance by 600 steps per week (week 1: 4600 steps per day), following indications from previous research with adolescents using mobile apps [88].

To facilitate the compliance of the adolescents with the recommendations, the steps were converted into kilometers, as STRAVA records the distance traveled in kilometers. In this respect, 4600 steps correspond to 2.94 km and 10,000 steps to 6.40 km, considering that 1 km represents approximately 1565 steps in the adolescent population [89]. This mobile app was selected because it has been proven to increase physical activity [90] and because it includes numerous techniques for behavioral change [91].

At the end of the 10 weeks, the physical activity level, body composition, psychological state, and physical condition of all adolescents were reevaluated (postintervention measurements). Those adolescents in the EG who did not complete the minimum weekly distance were not excluded from the study, nor were those who did not complete the intervention.

The final study sample consisted of 50 adolescents (26 EG and 24 CG). The attrition rate of the research was 13% (4/26) in the EG and 20% (6/24) in the CG. The mean reasons for dropping out of the intervention were as follows: changing schools (n=3), not attending the postintervention measurements (n=5), and not wanting to continue in the research (n=2).

### Data Analysis

The normality of the variables was analyzed using the Shapiro-Wilk test, as well as kurtosis and skewness. As the variables followed a normal distribution, parametric tests were used for the analysis. The Levene test was used to assess the homogeneity between the EG and CG in the study variables. A mixed model ANOVA was carried out to analyze intragroup differences in physical activity, kinanthropometric and body composition, psychological state, and physical condition. Subsequently, 3 analyses of covariance were carried out to analyze the differences in the study variables when gender, age, and distance covered with the app were included. For the analysis of change, an ANOVA was performed comparing the difference between pre and post EG with respect to the difference between pre and post CG. Two analyses of covariance were subsequently performed with the covariate gender and age. Partial eta squared (η^2^) was used to calculate the effect size and was defined as small: ES≥0.10; moderate: ES≥0.30; large: ES≥1.2; or very large: ES≥2.0, with an error of *P*<.05 [92]. A value of *P*<.05 was set to determine statistical significance. The statistical analysis was performed with the SPSS statistical package (version 25.0; SPSS Inc).

### Ethical Considerations

Before the start of the study, the institutional ethics committee of the Catholic University of Murcia approved the research design in accordance with the World Medical Association (CE022102). Informed consent was obtained from all individual participants included in the study.

## Results

The sample flow diagram showing the final participants in the CG and EG can be found in Figure 1.

### Normality and Homogeneity Tests

The results of the normality test are presented in Table 1.

Levene test for homogeneity showed that there was homogeneity between the EG and CG in physical activity (*P*=.54), body mass (*P*=.11), BMI (*P*=.93), waist girth (*P*=.34), hip girth (*P*=.97), waist-height ratio (*P*=.06), corrected arm girth (*P*=.54), corrected thigh girth (*P*=.43), corrected calf girth (*P*=.78), fat mass (*P*=.17), muscle mass (*P*=.41), sum of skinfold in 3 areas (*P*=.30), AMD (*P*=.55), life satisfaction (*P*=.07), competence (*P*=.91), autonomy (*P*=.57), relatedness (*P*=.65), maximal oxygen uptake (*P*=.46), handgrip right hand (*P*=.32), handgrip left hand (*P*=.57), sit and reach (*P*=.15), CMJ (*P*=.09), 20-m sprint (*P*=.35), curl up (*P*=.39), and push up (*P*=.05).

**Table 1:** Results of the normality analysis for control and experimental group variables.

	Experimental group, *P* value	Control group, *P* value
Physical activity	.78	.46
Body mass	.10	.20
BMI	.11	.06
Waist girth	.08	.53
Hip girth	.10	.18
Waist-height ratio	.07	.18
Corrected arm girth	.22	.06
Corrected thigh girth	.97	.62
Corrected calf girth	.30	.95
Fat mass	.91	.98
Muscle mass	.08	.08
Sum of skinfold in 3 areas	.69	.80
Adherence to the Mediterranean diet	.29	.52
Life satisfaction	.20	.21
Competence	.52	.21
Autonomy	.11	.81
Relatedness	.34	.75
Maximal oxygen uptake	.42	.05
Handgrip right hand	.07	.19
Handgrip left hand	.12	.75
Sit and reach	.61	.15
Countermovement jump	.99	.08
20-m sprint	.09	.59
Curl up	.35	.06
Push up	.30	.38

### Distance Covered With the App

The distance traveled by the EG adolescents using the apps was recorded weekly. The average distance traveled was 76.44 (SD 39.10) km over the 10 weeks of intervention, with the maximum distance traveled by one of the adolescents being 186.2 km, while the minimum was 14.43 km. It is worth noting that the final distance to be completed with the program was 139.97 km over the 10 weeks of intervention, and only 12% (3/26) adolescents reached or exceeded this distance. In fact, 54% (14/26) of the adolescents did not continue to use the app systematically after the sixth week.

### Physical Activity Level, AMD, and Psychological State

Table 2 shows the pre- and postintervention differences in the EG and CG for physical activity level, AMD, and psychological variables. The results showed only a significant decrease in competence in the adolescents in the CG (mean difference: 3.08; *P*=.03), with no significant changes in the other variables.

The influence of the covariates gender, age, and distance covered with the app on the level of physical activity, AMD, and psychological variables can be found in Table 3. Results showed influence of gender and age on AMD (gender: *P*=.04; age: *P*=.05), competence (gender: *P*=.04; age: *P*=.04) and relatedness (gender: *P*=.05; age: *P*=.04) of CG adolescents. No significant differences were found for the covariate distance covered with the app.

**Table 2:** Differences between the pre- and postintervention measurements in the experimental group (EG) and control group (CG) groups of adolescents who are overweight or obese in physical activity, adherence to the Mediterranean diet (AMD), and psychological state.

Variables and time point			Before intervention, mean (SD)		After intervention, mean (SD)		Prepost difference, mean (SD)		*F* test (*df*)		*P* value		95% CI difference		η^2^
**Physical activity**															
	EG	2.51 (0.74)		2.62 (0.68)		–0.11 (0.07)		2.430 (1)		.13		–0.248 to 0.031		0.048	
	CG	2.63 (0.64)		2.69 (0.55)		–0.06 (0.07)		0.706 (1)		.41		–0.207 to 0.085		0.015	
**AMD**															
	EG	6.73 (2.16)		6.46 (2.45)		0.27 (0.39)		0.489 (1)		.49		–0.505 to 1.044		0.010	
	CG	7.21 (2.43)		6.42 (2.62)		0.79 (0.40)		3.901 (1)		.05		–0.014 to 1.598		0.075	
**Life satisfaction**															
	EG	17.00 (3.14)		16.88 (2.90)		0.12 (0.70)		0.027 (1)		.87		–1.296 to 1.527		0.001	
	CG	18.46 (4.40)		17.21 (5.21)		1.25 (0.73)		2.926 (1)		.09		–0.219 to 2.719		0.094	
**Competence**															
	EG	24.54 (96.18)		23.85 (5.05)		0.69 (1.31)		0.281 (1)		.60		–1.933 to 3.317		0.006	
	CG	26.83 (5.98)		23.75 (9.40)		3.08 (1.36)		5.148 (1)		.03		0.351 to 5.816		0.097	
**Autonomy**															
	EG	23.96 (5.71)		23.88 (5.64)		0.07 (1.70)		0.002 (1)		.96		–3.338 to 3.491		0.001	
	CG	25.75 (5.03)		24.08 (8.67)		1.67 (1.77)		0.889 (1)		.35		–1.887 to 5.221		0.018	
**Relatedness**															
	EG	24.15 (6.70)		24.54 (6.33)		–0.39 (1.35)		0.082 (1)		.78		–0.385 to 1.347		0.002	
	CG	23.13 (5.98)		20.42 (8.58)		2.71 (1.40)		3.734 (1)		.06		2.708 to 1.402		0.072	

**Table 3:** Influence of the covariates gender, age, and distance covered with app in the physical activity level, adherence to the Mediterranean diet (AMD), and psychological state.

Variables and time point		App use × gender					App use × age					App use × distance covered with app			
		*F* test (*df*)	*P* value	95% CI difference	η^2^	*F* test (*df*)		*P* value	95% CI diff	η^2^	*F* test (*df*)		*P* value	95% CI difference	η^2^
**Physical activity**															
	EG^a^	1.913 (1)	.17	–0.247 to 0.046	0.039	1.006 (1)		.32	–0.211 to 0.070	0.021	0.157 (1)		.69	–0.183 to 0.123	0.003
	CG^b^	0.842 (1)	.36	–0.222 to 0.083	0.018	1.973 (1)		.17	–0.249 to 0.044	0.040	—^c^		—	—	—
**AMD**															
	EG	0.196 (1)	.66	–0.626 to 0.980	0.004	0.273 (1)		.60	–0.599to 1.020	0.006	3.120 (1)		.08	–0.115 to 1.766	0.062
	CG	4.576 (1)	.04	0.053 to 1.730	0.089	4.148 (1)		.05	0.010 to 1.701	0.081	—		—	—	—
**Life satisfaction**															
	EG	0.010 (1)	.92	–1.535 to 1.388	<0.001	0.012 (1)		.91	–1.545 to 1.385	0.000	0.120 (1)		.73	–1.879 to 1.327	0.003
	CG	3.682 (1)	.06	–0.071 to 2.980	0.073	3.699 (1)		.06	–0.067 to 2.991	0.073	—		—	—	—
**Competence**															
	EG	0.295 (1)	.59	–2.004 to 3.488	0.006	0.241 (1)		.63	–2.082 to 3.426	0.005	0.564 (1)		.46	–1.882 to 4.123	0.12
	CG	4.521 (1)	.04	0.163 to 5.896	0.088	4.723 (1)		.04	0.231 to 5.980	0.091	—		—	—	—
**Autonomy**															
	EG	0.190 (1)	.89	–3.321 to 3.815	<0.001	0.000 (1)		.99	–3.609 to 3.551	0.000	0.035 (1)		.85	–3.550 to 4283	0.001
	CG	0.642 (1)	.43	–2.241 to 5.206	0.013	0.920 (1)		.34	–1.956 to 5.519	0.019	—		—	—	—
**Relatedness**															
	EG	0.210 (1)	.65	–3.460 to 2.176	0.004	0.273 (1)		.60	–3.546 to 2.083	0.006	0.137 (1)		.71	–3.678 to 2.535	0.003
	CG	4.176 (1)	.047	0.046 to 5.929	0.082	4.459 (1)		.04	0.146 to 6.022	0.087	—		—	—	—

^a^EG: experimental group.

^b^CG: control group.

^c^Not applicable.

### Kinanthropometric and Body Composition Measurements

Table 4 shows the differences in kinanthropometric and body composition variables between pre- and postintervention measurements in the CG and EG. The differences were significant in the EG, with an increase in corrected arm girth (mean difference: –0.46; *P*=.05), as well as in the CG, with an increase in hip girth (mean difference: –1.37; *P*=.05), corrected thigh girth (mean difference: –1.28; *P*=.04), and muscle mass (mean difference –0.87; *P*=.04).

The covariate gender showed an effect on the variables corrected arm girth (*P*=.04) of the EG, as well as the corrected thigh girth (*P*=.04) of the CG. No significant differences were found for the covariate age nor for the covariate distance covered (Table 5).

**Table 4:** Differences between the pre- and postintervention measurements in the experimental group (EG) and control group (CG) of adolescents who are overweight or obese in the kinanthropometric and body composition measurements.

Variables and time point			Before intervention, mean (SD)		After intervention, mean (SD)		Prepost difference, mean (SD)		*F* test (*df*)		*P* value		95% CI difference		η^2^
**Body mass (kg)**															
	EG	75.78 (16.83)		75.60 (17.62)		0.18 (0.43)		0.168 (1)		.68		–0.691 to 1.045		0.003	
	CG	70.21 (10.78)		70.74 (11.08)		–0.53 (0.45)		1.386 (1)		.25		–1.433 to 0.375		0.028	
**BMI (kg/m^2^)**															
	EG	27.76 (3.51)		27.67 (3.79)		0.09 (0.19)		0.235 (1)		.63		–0.291 to 0.475		0.005	
	CG	27.71 (3.18)		27.52 (2.89)		0.18 (0.20)		0.855 (1)		.36		–0.215 to 0.582		0.018	
**Waist girth (cm)**															
	EG	82.11 (10.39)		81.55 (11.09)		0.57 (0.68)		0.701 (1)		.41		–0.796 to 1.929		0.015	
	CG	82.71 (8.66)		83.19 (7.50)		–0.48 (0.74)		0.419 (1)		.52		–1.957 to 1.005		0.009	
**Hip girth (cm)**															
	EG	104.93 (8.57)		104.96 (9.15)		–0.02 (0.62)		0.001 (1)		.97		–1.269 to 1.224		0.001	
	CG	99.65 (7.92)		101.02 (7.23)		–1.37 (0.67)		4.157 (1)		.05		–2.728 to –0.017		0.083	
**Waist-height**															
	EG	0.50 (0.04)		0.49 (0.05)		0.00 (0.01)		1.070 (1)		.31		–0.004 to 0.013		0.023	
	CG	0.52 (0.07)		0.52 (0.06)		0.00 (0.01)		0.007 (1)		.94		–0.009 to 0.009		0.001	
**Corrected arm girth (cm)**															
	EG	23.55 (2.29)		24.01 (2.53)		–0.46 (0.22)		4.185 (1)		.05		–0.911 to –0.007		0.083	
	CG	23.55 (2.75)		23.86 (2.60)		–0.31 (0.24)		1.635 (1)		.21		–0.803 to 0.179		0.034	
**Corrected thigh girth (cm)**															
	EG	43.91 (5.68)		44.82 (5.47)		–0.91 (0.57)		2.607 (1)		.11		–2.052 to 0.225		0.054	
	CG	42.69 (4.81)		43.97 (4.00)		–1.28 (0.62)		4.321 (1)		.04		–2.517 to –0.040		0.054	
**Corrected calf girth (cm)**															
	EG	30.77 (6.62)		30.25 (3.67)		0.52 (0.94)		0.312 (1)		.58		–1.364 to 2.411		0.007	
	CG	29.21 (3.73)		29.77 (3.30)		–0.56 (1.02)		0.301 (1)		.59		–2.611 to 1.493		0.007	
**Fat mass (%)**															
	EG	39.14 (10.28)		37.09 (8.85)		2.04 (1.25)		2.679 (1)		.11		–0.470 to 4.557		0.055	
	CG	36.64 (13.74)		37.33 (10.96)		–0.69 (1.36)		0.258 (1)		.61		–3.422 to 2.043		0.006	
**Muscle mass (kg)**															
	EG	21.59 (6.01)		22.18 (6.43)		–0.59 (0.37)		2.578 (1)		.12		–1.327 to 0.149		0.053	
	CG	20.95 (5.07)		21.82 (4.78)		–0.87 (0.40)		4.737 (1)		.04		–1.671 to –0.065		0.093	
**Summary of skinfold in 3 areas**															
	EG	93.27 (28.71)		87.29 (23.97)		5.98 (3.50)		2.922 (1)		.09		–1.062 to 13.023		0.060	
	CG	85.56 (35.56)		84.53 (26.47)		1.03 (3.80)		0.073 (1)		.79		–6.631 to 8.682		0.002	

**Table 5:** Influence of the covariates gender, age, and distance covered with app in the kinanthropometric and body composition measurements.

Variables and time point			App use × gender									App use × age									App use × distance covered with app						
			*F* test (*df*)		*P* value		95% CI difference		η^2^		*F* test (*df*)			*P* value		95% CI difference		η^2^		*F* test (*df*)			*P* value		95% CI difference		η^2^
**Body mass (kg)**																											
	EG^a^	0.051 (1)		.82		–0.803 to 1.006		0.001		0.028 (1)			.87		–0.829 to 0.979		0.001		0.392 (1)			.54		–0.684 to 1.302		0.008	
	CG^b^	0.910 (1)		.35		–1.391 to 0.496		0.019		0.798 (1)			.38		–1.362 to 0.525		0.017		—^c^			—		—		—	
**BMI (kg/m^2^)**																											
	EG	0.036 (1)		.85		–0.358 to 0.433		0.001		0.153 (1)			.70		–0.323 to 0.480		0.003		1.231 (1)			.27		–0.193 to 0.668		0.026	
	CG	1.400 (1)		.24		–0.170 to 0.656		0.029		0.910 (1)			.35		–0.220 to 0.618		0.019		—			—		—		—	
**Waist girth (cm)**																											
	EG	0.356 (1)		.55		–0.998 to 1.838		0.008		1.331 (1)			.26		–0.595 to 2.192		0.029		0.843 (1)			.36		–0.841 to 2.251		0.018	
	CG	0.155 (1)		.70		–1.854 to 1.249		0.003		0.984 (1)			.33		–2.274 to 0.773		0.021		—			—		—		—	
**Hip girth (cm)**																											
	EG	0.045 (1)		.83		–1.438 to 1.164		0.001		0.084 (1)			.77		–1.094 to 1.461		0.002		0.014 (1)			.91		–1.332 to 1.500		0.001	
	CG	3.070 (1)		.09		–2.660 to 0.185		0.064		5.439 (1)			.05		–3.012 to –0.220		0.088		—			—		—		—	
**Waist-height**																											
	EG	0.638 (1)		.43		–0.005 to 0.012		0.014		1.961 (1)			.17		–0.003 to 0.014		0.042		1.768 (1)			.19		–0.004 to 0.017		0.038	
	CG	0.083 (1)		.77		–0.008 to 0.011		0.002		0.111 (1)			.74		–0.011 to 0.008		0.002		—			—		—		—	
**Corrected arm girth (cm)**																											
	EG	4.378 (1)		.04		–0.963 to –0.180		0.089		2.768 (1)			.10		–0.844 to 0.080		0.058		2.914 (1)			.10		–0.948 to 0.078		0.061	
	CG	1.147 (1)		.29		–0.791 to 0.242		0.025		2.588 (1)			.12		–0.908 to 0.102		0.054		—			—		—		—	
**Corrected thigh girth (cm)**																											
	EG	1.962 (1)		.17		–2.018 to 0.362		0.042		3.130 (1)			.08		–2.216 to 0.143		0.065		2.471 (1)			.12		–2.303 to 0.284		0.052	
	CG	4.558 (1)		.04		–2.682 to –0.078		0.092		3.135 (1)			.08		–2.423 to 0.156		0.065		—			—		—		—	
**Corrected calf girth (cm)**																											
	EG	0.117 (1)		.73		–1.633 to 2.302		0.003		0.205 (1)			.65		–1.526 to 2.410		0.005		0.373 (1)			.55		–1.495 to 2.795		0.008	
	CG	0.099 (1)		.76		–2.487 to 1.817		0.002		0.188 (1)			.67		–2.614 to 1.689		0.004		—			—		—		—	
**Fat mass (%)**																											
	EG	2.105 (1)		.15		–0.736 to 4.524		0.045		2.658 (1)			.11		–0.500 to 4.745		0.056		2.196 (1)			.15		–0.755 to 4.960		0.047	
	CG	0.129 (1)		.72		–3.390 to 2.364		0.003		0.303 (1)			.59		–3.649 to 2.084		0.007		—			—		—		—	
**Muscle mass (kg)**																											
	EG	2.857 (1)		.10		–1.419 to 0.124		0.060		2.707 (1)			.11		–1.398 to 0.141		0.057		1.602 (1)			.21		–1.366 to 0.312		0.034	
	CG	3.639 (1)		.06		–1.643 to 0.045		0.075		3.869 (1)			.06		–1.662 to 0.020		0.079		—			—		—		—	
**Summary of skinfold in 3 areas**																											
	EG	2.092 (1)		.16		–2.069 to 12.610		0.044		3.789 (1)			.06		–0.243 to 14.249		0.078		2.774 (1)			.10		–1.385 to 14.611		0.058	
	CG	0.219 (1)		.64		–6.164 to 9.893		0.005		0.002 (1)			.96		–8.104 to 7.738		0		—			—		—		—	

^a^EG: experimental group.

^b^CG: control group.

^c^Not applicable.

### Physical Fitness Tests

Table 6 shows the differences in the fitness variables between pre- and postintervention measurements. In the EG an increase in curl-up (mean difference: –6.35; *P*=.02) and push-up (mean difference: –2.27; *P*=.04) was found after the intervention. No significant differences were found in the CG.

The influence of the covariates on the fitness variables is shown in Table 7. The covariate gender showed an effect on the variables curl-up (*P*=.04) and push-up (*P*=.04) of the EG. No significant differences were found for the covariate age, nor for the covariate distance covered.

**Table 6:** Differences between the pre- and postintervention measurements in the experimental group (EG) and control group (CG) of adolescents who are overweight or obese in the physical fitness variables.

Variables and time point			Before intervention, mean (SD)		After intervention, mean (SD)		Pre-post difference, mean (SD)		*F* test (*df*)		*P* value		95% CI difference		η^2^
**VO**_**2**_ **max** ^a^ **(mL/kg/min)**															
	EG	34.55 (3.43)		34.40 (4.08)		0.15 (0.40)		0.135 (1)		.72		–0.662 to 0.957		0.003	
	CG	36.56 (4.70)		36.59 (4.92)		–0.03 (0.41)		0.006 (1)		.94		–0.859 to 0.798		0.001	
**Handgrip right hand (kg)**															
	EG	26.93 (6.46)		27.55 (6.46)		–0.62 (0.99)		0.391 (1)		.54		–2.612 to 1.373		0.008	
	CG	26.03 (9.28)		25.42 (11.35)		0.61 (1.03)		0.353 (1)		.56		–1.461 to 2.686		0.007	
**Handgrip left hand (kg)**															
	EG	25.32 (6.79)		25.29 (6.80)		0.03 (0.60)		0.003 (1)		.96		–1.175 to 1.237		0.001	
	CG	24.72 (7.52)		25.15 (8.86)		–0.44 (0.62)		0.491 (1)		.49		–1.693 to 0.818		0.010	
**Sit-and-reach (cm)**															
	EG	16.29 (9.84)		17.67 (10.59)		–1.39 (0.82)		2.879 (1)		.10		–3.026 to 0.257		0.058	
	CG	13.22 (7.53)		14.78 (7.58)		–1.57 (0.87)		3.254 (1)		.08		–3.311 to 0.180		0.065	
**Countermovement jump (cm)**															
	EG	17.93 (5.32)		18.49 (6.58)		–0.56 (1.08)		0.271 (1)		.61		–2.727 to 1.605		0.006	
	CG	18.27 (7.60)		19.45 (7.93)		–1.19 (1.12)		1.122 (1)		.30		–3.442 to 1.067		0.023	
**20 m** **sprint**															
	EG	4.05 (0.96)		3.78 (1.20)		0.27 (0.16)		2.880 (1)		.10		–0.049 to 0.585		0.057	
	CG	4.17 (1.10)		4.25 (0.63)		–0.08 (0.16)		0.228 (1)		.64		–0.408 to 0.252		0.005	
**Curl-up (repetitions)**															
	EG	17.23 (11.28)		23.58 (9.29)		–6.35 (2.51)		6.382 (1)		.02		–11.397 to –1.295		0.117	
	CG	18.17 (12.93)		21.71 (12.86)		–3.54 (2.62)		1.835 (1)		.18		–8.799 to 1.716		0.037	
**Push-up (repetitions)**															
	EG	1.88 (4.48)		4.15 (5.81)		–2.27 (1.05)		4.698 (1)		.04		–4.377 to –0.162		0.093	
	CG	4.86 (6.47)		4.77 (6.24)		0.09 (1.14)		0.006 (1)		.94		–2.200 to 2.382		0.001	

^a^VO_2_max: maximal oxygen uptake.

**Table 7:** Influence of the covariates gender, age, and distance covered with app in the physical fitness variables.

Variables and time point			App use × gender								App use × age								App use × distance covered with app						
			*F* test (*df*)		*P* value		95% CI difference		η^2^		*F* test (*df*)		*P* value		95% CI difference		η^2^		*F* test (*df*)		*P* value		95% CI difference		η^2^
**VO**_**2**_ **max** ^a^ **(mL/kg/min)**																									
	EG^b^	0.199 (1)		.66		–0.917 to 0.585		0.005		0.012 (1)		.91		–0.793 to 0.886		0.000		1.441 (1)		.24		–0.359 to 1.408		0.035	
	CG^c^	0.611 (1)		.44		–0.472 to 1.068		0.015		0.031 (1)		.86		–0.786 to 0.936		0.001		—^d^		—		—		—	
**Handgrip right hand (kg)**																									
	EG	0.638 (1)		.43		–2.895 to 1.249		0.013		0.045 (1)		.83		–2.259 to 1.826		0.001		0.400 (1)		.53		–3.006 to 1.568		0.008	
	CG	0.601 (1)		.44		–1.329 to 2.995		0.013		0.028 (1)		.87		–1.956 to 2.308		0.001		—		—		—		—	
**Handgrip left hand (kg)**																									
	EG	0.003 (1)		.96		–1.229 to 1.295		0.001		0.067 (1)		.80		–1.095 to 1.418		0.001		0.153 (1)		.70		–1.639 to 1.106		0.003	
	CG	0.452 (1)		.51		–1.757 to 0.877		0.010		0.788 (1)		.38		–1.891 to 0.733		0.016		—		—		—		—	
**Sit-and-reach (cm)**																									
	EG	1.977 (1)		.17		–2.915 to 0.517		0.041		2.133 (1)		.15		–2.947 to 0.469		0.044		2.806 (1)		.10		–3.432 to 0.314		0.058	
	CG	3.797 (1)		.06		–3.609 to 0.059		0.076		3.647 (1)		.06		–3.553 to 0.094		0.073		—		—		—		—	
**Countermovement jump (cm)**																									
	EG	0.120 (1)		.73		–2.646 to 1.869		0.003		0.273 (1)		.60		–2.862 to 1.682		0.006		0.179 (1)		.67		–3.011 to 1.963		0.004	
	CG	1.377 (1)		.25		–3.731 to 0.982		0.028		0.961 (1)		.33		–3.528 to 1.216		0.020		—		—		—		—	
**20-m sprint**																									
	EG	3.260 (1)		.08		–0.034 to 0.627		0.065		1.807 (1)		.19		–0.109 to 0.548		0.037		1.274 (1)		.27		–0.159 to 0.565		0.026	
	CG	0.408 (1)		.53		–0.454 to 0.235		0.009		0.023 (1)		.88		–0.369 to 0.317		0.000		—		—		—		—	
**Curl-up (repetitions)**																									
	EG	4.492 (1)		.04		–10.657 to –0.278		0.087		3.692 (1)		.06		–9.801 to 0.225		0.073		3.319 (1)		.08		–10.972 to 0.543		0.066	
	CG	2.785 (1)		.10		–9.910 to 0.923		0.056		4.042 (1)		.05		–10.463 to 0.004		0.079		—		—		—		—	
**Push-up (repetitions)**																									
	EG	4.292 (1)		.04		–4.482 to –0.063		0.087		3.764 (1)		.06		–4.359 to 0.082		0.077		3.326 (1)		.08		–4.569 to 0.227		0.069	
	CG	0.006 (1)		.94		–2.322 to 2.512		0.001		0.003 (1)		.96		–2.495 to 2.368		0.000		—		—		—		—	

^a^VO_2_max: maximal oxygen consumption.

^b^EG: experimental group.

^c^CG: control group.

^d^Not applicable.

### Analysis of Change Between CG and EG

Multimedia Appendix 1 shows an analysis of pre-post differences found in the EG and CG. It should be noted that no significant differences were found with the use of the app, nor after the inclusion of the covariates gender and age.

## Discussion

### Principal Findings

The main results of the research showed that a large proportion of the adolescents did not continue using the app after the first 6 weeks. Some changes were observed in the study variables, including a decrease in competence in the CG but not in the EG; an increase in corrected arm girth in the EG and in hip girth, corrected thigh girth, and muscle mass in the CG; and an improvement in curl-up repetitions and push-up repetitions in the EG. The covariates also showed an effect on the study variables, mainly gender and age, but not the distance covered with the app. However, despite the results obtained, the changes produced in the EG were not significantly different from those produced in the CG.

The present investigation originated from previous research that had been carried out in the school setting with adolescents who are overweight and obese and that had been effective, mainly on body composition and physical condition. In this regard, school-based HIIT interventions had shown effectiveness on body composition and cardiorespiratory fitness [42,43,93]. These interventions were characterized by being carried out in short periods of time and at high intensity, even allowing to obtain benefits in cardiac and blood parameters, being very beneficial for the health of adolescents [93,94]. In addition to interventions based on HIIT, other interventions based on the accumulation of minutes of physical activity have also been carried out in the school environment, restructuring the organization of classes, increasing the possible physical activity during class, or using breaks between classes. These have also reported benefits on fitness, physical activity level, body composition, and psychological state [95,96]. However, given the limited number of hours of physical education in the school context and the large amount of content that needs to be addressed in the classroom, such interventions have limited effects as they cannot be sustained in the long term [44,45]. So, resources are needed to promote the practice of physical activity outside of school hours. In this context, previous literature has shown mixed results in promoting out-of-school physical activity, with interventions that have led to increased physical activity among adolescents but with others that do not perceive benefits in physical activity levels or psychological variables, such as motivation or competence [97,98]. One of the main reasons for the lack of conclusive results is that after-school physical activity has the problem of physical education teachers’ lack of control over what is being done [99]. In this sense, mobile devices allow tracking of the physical activity performed in the out-of-school environment, so these apps have proven to be a useful alternative to promote physical activity in out-of-school hours [100,101].

The first objective of this research was to determine the changes produced by a 10-week intervention promoted by the physical education school subject using step tracker mobile apps in out-of-school hours on physical activity, AMD, body composition, and physical condition of adolescents who are overweight and obese. The results showed that the EG improved their performance in the curl-up and push-up tests, while the CG showed no significant differences in any fitness test. These results are in line with previous research, in which the use of mobile apps for walking improved the physical fitness of adolescents aged between 12 and 16 years [19,102]. Following previous research in this area, a possible explanation for the improvement obtained in the curl-up test would be that the abdominal muscles are activated when walking, increasing their resistance [102]. Regarding the significant improvement in the push-up test, this could be because the initial physical fitness level of the participants was very low, with an average of 2 repetitions performed in this test, so the improvement achieved with the training program could be sufficient to increase performance in certain physical fitness tests [88,103]. However, in the specific case of the present research, no significant improvements in physical activity were observed, nor was there a direct influence of the distance traveled with the use of the app in these variables, so it would be difficult to assume that the changes are due to the intervention. Therefore, another possible explanation would be the maturational process in which the adolescents are immersed since, as the characteristics of the sample show, most of them had already passed the APHV [104]. Around and behind the APHV, hormonal and physical changes occur that lead to changes in body composition and athletic performance [105], with boys increasing their strength and production of hormones, such as testosterone, to a greater extent [105]. This becomes even more relevant when considering that the covariate gender did have an influence on performance in both physical tests, with boys performing better, as was the case in previous research [106]. However, the small sample size makes it difficult to analyze differences according to gender and maturational status, so future studies should address both questions. This would allow us to discover whether this type of intervention program is effective in improving the physical fitness of adolescents who are overweight or obese, or whether it is the gender differences or the effect of the maturational process that led to the changes.

It is important to highlight that the EG did not show significant improvements in the performance of the other fitness tests (20-m shuttle run test, sit-and-reach, handgrip strength, CMJ, and 20-m sprint), which could be because improvements in these tests require a moderate to vigorous intensity of physical practice, as shown in previous scientific literature [107]. The intervention program used included only walking activities, so it is likely that the intensity at which they performed the training was a determining factor in the lack of benefits [108]. In addition, adherence to the training program was low, with >50% (n=14) of the adolescents dropping out of regular training after the sixth week and <12% (n=3) meeting targets at the end of 10 weeks. This makes the intervention program too short, which may influence the benefits obtained, as previous interventions show that programs with a minimum of 10 weeks are the ones that truly lead to significant changes [19]. Therefore, future research should place more emphasis on training parameters, such as volume and intensity, as these are relevant aspects in obtaining improvements with aerobic training, as shown in previous scientific literature [109,110]. Regarding body composition, the results showed no significant changes in the EG in the variables related to fat mass. The absence of changes in the present investigation could be because the adolescents were not instructed on the intensity at which the walking should be performed, so it is likely that a moderate to vigorous intensity that would produce changes in body composition was not reached [108,110]. This is similar to previous research that compared changes in body composition in adolescents after participating in high-, medium-, and low-intensity aerobic exercise programs, with adolescents in the low-intensity program having the smallest gains in body composition [111].

However, in the variables related to muscle mass, significant changes were observed in both the EG and CG, as an increased corrected arm girth in the EG and corrected thigh girth and muscle mass in the CG. Given that the intervention did not include strength exercises and that those improvements occurred in both groups, one explanation for the increase in muscle mass-related variables would be the increase in the production of steroid hormones that occurs during adolescence [105]. This favors the development of muscle mass and the gain in strength [71]. AMD showed no significant differences between pre- and postintervention measurements in any of the groups analyzed. These results are in line with previous research on adolescents, in which the use of mobile physical activity apps had no influence on AMD [19]. The rationale for including AMD in a physical activity intervention lay in the fact that previous research had shown that those who were more physically active had greater AMD [26,27]. However, in this research, no significant improvement in physical activity level was achieved, which would hinder the improvement of other healthy habits, such as AMD. This could be because the apps currently available on the market for physical activity do not include nutritional content, making it difficult to achieve benefits in this area, as the interventions carried out specifically with mobile nutrition apps do show significant improvements [112]. It is worth noting that the covariates gender and age significantly affected the AMD of the CG adolescents. This is consistent with previous research that found significant differences in AMD between adolescent boys and girls [112], as well as a decrease in AMD adherence with age [27]. Future research should consider mobile apps that include physical activity and nutrition together, as the benefits obtained could be greater for adolescents, as shown in physical exercise programs combined with nutrition [107,112].

The results obtained allow us to reject the first research hypothesis, as it was expected that the promotion of the use of mobile apps from the school subject of physical education would lead to significant benefits in the level of physical activity, favoring changes in body composition, greater AMD, and an improvement in the physical condition of this population. However, the results show that the adolescents of the EG did not achieve significant improvements in the level of physical activity, so no changes were found in most of the variables of body composition, AMD, or physical fitness. Therefore, the proposed program may not be effective, probably due to a lack of adherence, as most of the adolescents stopped regular physical activity after the sixth week. Future research is needed to draw conclusions in this area.

The second aim of this research was to analyze the changes achieved by the 10-week intervention on the psychological state of adolescents who are overweight and obese. The results showed a significant decrease in the competence variable in the CG. Previous research has shown that adolescents with less physical activity showed lower scores on the competence variable [107]. Furthermore, it is worth noting that no changes in the perception of competence were found in the adolescents belonging to the EG. This could be because the intervention was not sufficient for adolescents to perceive that they had increased their physical activity. This may be due to a lack of compliance by participants. Therefore, given these preliminary results, future research is needed to analyze the relationship between the use of mobile apps and the psychological state of the adolescents who are overweight and obese, as well as the relationship with other variables, such as motivation or engagement.

The inclusion of the covariates gender and age showed significant effects on the competence and relatedness of CG adolescents. This is an aspect to be considered, as previous research has shown that physical activity practice is lower in adolescent girls [113], as well as in older adolescents [114]. These groups place greater importance on physical activity to promote social interaction compared to boys and younger adolescents [113,115]. In addition, during adolescence, body image becomes particularly relevant, even altering healthy behaviors, such as nutrition and physical activity [116], with women being particularly affected in this respect [116]. Despite the results obtained, future research with a larger sample is needed to perform a separate analysis according to gender and age to know the real effect of mobile apps on the psychological state of adolescents who are overweight and obese.

The results obtained allow us to reject the second research hypothesis, which stated that increased physical activity has a positive effect on the psychological state of adolescents who are overweight and obese, because only significant decreases were observed in the scores of the psychological variables of the CG, with no changes in the EG. Furthermore, future research should give special relevance to the analysis of differences in psychological state between men and women of different ages who are overweight and obese and who use mobile apps for physical activity.

Although the present research is pioneering in the promotion of physical activity in the adolescent population that is overweight or obese during out-of-school hours by means of mobile apps, it is not without limitations. First, the sample size was small, which made it impossible to divide it into gender or maturity groups. In addition, the small sample of adolescents who completed the intervention in its entirety precludes comparison of actual effects with the CG. Although AMD was assessed, caloric intake was not monitored, which could be a determining factor in the changes achieved in the body composition of the adolescents. The level of physical activity was assessed subjectively by the PAQ-A questionnaire but was not assessed objectively by means of accelerometry. Similarly, psychological state was assessed by validated questionnaires, but the inclusion of other techniques, such as interviews, in future research could provide more information. Although the total distance traveled with the use of the app was measured, as well as the steps taken by each adolescent, the information that the adolescents walked before starting the intervention was not recorded, so it is not known whether there was an improvement in the daily steps taken. Finally, BMI ≥25 kg/m^2^ has been used to define adolescents who are overweight and obese instead of WHO or National Center for Health Statistics growth charts, as it is a simple method [55,56] and is consistent with growth charts when measured accurately [52,55], as was done in the present investigation. However, future research could replicate this study using WHO or National Center for Health Statistics growth charts, especially if applied to a population of children rather than adolescents. All the above limitations affect the generalizability of the study results to the population of adolescents who are overweight and obese.

As for the practical applications derived from this study, it is observed that in adolescents who are overweight and obese, going for a walk in their free time offers benefits on physical condition and psychological state, or at least prevents a worsening of these variables. However, the proposal put forward does not seem to be sufficient to obtain improvements in body composition in this population, which could be because adherence to the intervention with mobile apps was reduced, and from the sixth week onward, few adolescents complied with the training plan. Therefore, the results obtained are relevant for physical education teachers and for the health field because mobile physical activity apps could be a useful element for the promotion of active time in adolescents who are overweight or obese, although future research is needed to provide more scientific evidence in this regard and to increase adherence to these programs. This may require the use of other mobile apps in which gamification is a major part of it, as in previous research, it has been observed that this aspect favors adherence [19] and controls aspects, such as intensity or training volume, which may be relevant to achieve benefits.

### Conclusions

The findings of this study affirm that a 10-week program consisting of the use of mobile apps by adolescents who are overweight and obese to practice physical activity outside school hours is not effective in improving the level of physical activity in this population, and therefore no improvements in body composition, fitness, or psychological state were found. The lack of benefits could be because adherence to the program was very low, with >50% (14/26) of the adolescents failing to meet the objectives set after the sixth week of the intervention and <12% (3/26) meeting targets at the end of 10 weeks. Therefore, future research is needed to provide more evidence in this area and to select the optimal mobile apps to achieve benefits in this population.

## Appendix

Multimedia Appendix 1 Changes between the intervention and control groups.

Multimedia Appendix 2 CONSORT (Consolidated Standards of Reporting Trials) checklist.

## Abbreviations

AMD: adherence to the Mediterranean diet
APHV: age at peak height velocity
BPNS: Basic Psychological Needs Scale
CG: control group
CMJ: countermovement jump
CONSORT: Consolidated Standards of Reporting Trials
EG: experimental group
HIIT: high-intensity interval training
ISAK: International Society for the Advancement of Kinanthropometry
KIDMED: Mediterranean Diet Quality Index
PAQ-A: Physical Activity Questionnaire for Adolescents
SWLS: Satisfaction With Life Scale
TEM: technical errors of measurement
WHO: World Health Organization

## Data Availability

The datasets generated during and analyzed during this study are available from the corresponding author on reasonable request. The measurement protocol was registered before the start of the study at ClinicalTrials.gov (NCT06089876).
