# Supplementary material for: Effects of 10 Weeks of Walking With Mobile Step-Tracking Apps on Body Composition, Fitness, and Psychological State in Adolescents Who Are Overweight and Obese: Randomized Controlled Trial
Source: J Med Internet Res. 2024 Dec 10;26:e55243. doi: 10.2196/55243 (PMC11668985; doi:10.2196/55243)
Supplement: Multimedia Appendix 1 [file jmir_v26i1e55243_app1.docx]

Table 7. Changes between the intervention and control groups.

| Variable | App use | | | | | App use * Gender | | | | App use * Age | | | |
| --- | --- | --- | --- | --- | --- | --- | --- | --- | --- | --- | --- | --- | --- |
|  | Pre-Post EG –  Pre-post CG | F | *P* | 95%CI diff. | η2 | F | *P* | 95%CI diff. | η2 | F | *P* | 95%CI diff. | η2 |
| Physical activity | -0.05±0.11 | 0.207 | .65 | -0.270; 0.171 | 0.005 | 0.071 | .79 | -0.267;  0.204 | 0.002 | 0.081 | .78 | -0.203;  0.270 | 0.002 |
| Body Mass (kg) | 0.86±0.65 | 1.724 | .20 | -0.463;  2.176 | 0.042 | 0.872 | .36 | -0.751;  2.038 | 0.022 | 1.703 | .20 | -0.521; 2.409 | 0.043 |
| BMI (kg/m^2^) | -0.09±0.31 | 0.075 | .79 | -0.716;  0.545 | 0.002 | 0.560 | .46 | -0.897;  0.413 | 0.015 | 0.008 | .93 | -0.731; 0.668 | 0.000 |
| Waist girth (cm) | 1.37±1.16 | 1.405 | .24 | -0.968;  3.709 | 0.035 | 0.668 | .42 | -1.475;  3.473 | 0.017 | 3.305 | .08 | -0.255; 4.751 | 0.080 |
| Hips girth (cm) | 1.35±1.04 | 1.679 | .20 | -0.757;  3.457 | 0.041 | 1.013 | .32 | -1.128;  3.357 | 0.026 | 4.009 | .06 | -0.024; 4.452 | 0.095 |
| Waist/height | 0.06±0.01 | 0.602 | .44 | -0.009;  0.020 | 0.015 | 0.199 | .66 | -0.012;  0.019 | 0.005 | 2.181 | .15 | -0.004; 0.026 | 0.054 |
| Corrected arm girth (cm) | -0.17±0.37 | 0.205 | .65 | -0.915;  0.581 | 0.005 | 0.315 | .58 | -1.020;  0.577 | 0.008 | 0.077 | .78 | -0.691; 0.912 | 0.002 |
| Corrected thigh girth (cm) | 0.38±0.89 | 0.182 | .67 | -1.426;  2.189 | 0.005 | 0.474 | .50 | -1.264;  2.565 | 0.012 | 0.032 | .86 | -1.827; 2.178 | 0.001 |
| Corrected calf girth (cm) | 1.39±1.61 | 0.751 | .39 | -1.859;  4.645 | 0.019 | 0.287 | .60 | -2.534;  4.355 | 0.007 | 0.356 | .56 | -2.543; 4.667 | 0.009 |
| Fat mass (%) | 3.01±2.05 | 2.158 | .15 | -1.134;  7.148 | 0.052 | 1.524 | .23 | -1.725;  7.115 | 0.039 | 2.344 | .13 | -1.118; 8.059 | 0.058 |
| Muscle mass (kg) | 0.30±0.60 | 0.246 | .62 | -0.912;  1.505 | 0.006 | 0.113 | .74 | -1.076;  1.505 | 0.003 | 0.163 | .69 | -1.075; 1.611 | 0.004 |
| Sum of 3 skinfolds | 5.09±5.70 | 0.797 | .38 | -6.443;  16.624 | 0.020 | 0.309 | .58 | -8.860;  15.565 | 0.008 | 1.736 | .20 | -4.391; 20.767 | 0.044 |
| AMD | -0.32±0.61 | 0.274 | .60 | -1.560;  0.918 | 0.007 | 0.565 | .46 | -1.803;  0.827 | 0.015 | 0.262 | .61 | -1.726; 1.029 | 0.007 |
| Life satisfaction | -0.62±0.85 | 0.532 | .47 | -2.328;  1.094 | 1.094 | 0.13 | .45 | -2.521;  1.139 | 0.015 | 0.409 | .53 | -2.503; 1.301 | 0.011 |
| Competence | -0.48±1.67 | 0.082 | .78 | -.3.848;  2.896 | 2.896 | 0.002 | .92 | -3.776;  3.416 | 0.000 | 0.014 | .91 | -3.488; 3.927 | 0.000 |
| Autonomy | 0.50±2.31 | 0.046 | .83 | -4.181;  5.171 | 5.171 | 0.001 | .62 | -3.708;  6.183 | 0.007 | 0.180 | .67 | -4.092; 6.261 | 0.005 |
| Relatedness | -2.11±1.58 | 1.778 | .19 | -5.311;  1.091 | 1.091 | 0.044 | .19 | -5.674;  1.171 | 0.045 | 2.540 | .12 | -6.289; 0.749 | 0.063 |
| VO2max (ml/kg/min) | 0.08±0.59 | 0.017 | .90 | -1.121;  1.276 | 0.000 | 0.791 | .38 | -1.637;  0.638 | 0.020 | 0.074 | .79 | -1.494; 1.139 | 0.002 |
| Handgrip right hand (kg) | -0.95±0.95 | 1.005 | .32 | -2.871;  0.968 | 0.025 | 0.541 | .47 | -2.786;  1.301 | 0.140 | 0.502 | .48 | -2.873; 1.383 | 0.013 |
| Handgrip left hand (kg) | -0.35±0.95 | 0.135 | .72 | -2.262;  1.567 | 0.003 | 0.135 | .72 | -2.421;  1.676 | 0.004 | 0.058 | .81 | -2.379; 1.874 | 0.002 |
| Sit-and-reach (cm) | 0.66±1.36 | 0.232 | .63 | -2.102;  3.416 | 0.006 | 0.412 | .53 | -2.008;  3.870 | 0.011 | 0.533 | .47 | -1.948; 4.145 | 0.014 |
| CMJ (cm) | 0.41±0.99 | 0.168 | .68 | -1.597;  2.407 | 0.004 | 0.068 | .80 | -1.863;  2.413 | 0.002 | 0.111 | .74 | -1.859; 2.591 | 0.003 |
| 20msprint (s) | 0.05±0.08 | 0.401 | .53 | -0.106;  0.202 | 0.010 | 0.493 | .49 | -0.107;  0.222 | 0.013 | 0.386 | .54 | -0.119; 0.224 | 0.010 |
| Curl-up (reps) | -2.55±3.83 | 0.441 | .51 | -10.301;  5.210 | 0.011 | 0.116 | .74 | -9.595;  6.831 | 0.003 | 0.027 | .87 | -9.177; 7.806 | 0.001 |
| Push-up (reps) | -1.67±1.55 | 1.164 | .29 | -4.800;  1.461 | 0.029 | 1.550 | .22 | -5.374;  1.281 | 0.039 | 1.470 | .23 | -5.540; 1.389 | 0.037 |

BMI: body mass index; sum: summatory; AMD: adherence to the Mediterranean diet; VO2 max: maximal oxygen consumption; CMJ: countermovement jump.
